# Affective norms for 1,586 polish words (ANPW): Duality-of-mind approach

**DOI:** 10.3758/s13428-014-0509-4

**Published:** 2014-10-08

**Authors:** Kamil K. Imbir

**Affiliations:** Maria Grzegorzewska Academy of Special Education (ASE), ul. Szczęśliwicka 40, 02-353 Warsaw, Poland

**Keywords:** Affective ratings, Valence, Arousal, Dominance, Origin, Source, Significance

## Abstract

This article presents valence/pleasantness, activity/arousal, power/dominance, origin, subjective significance, and source-of-experience norms for 1,586 Polish words (primarily nouns), adapted from the Affective Norms for English Words list (1,040 words) and from my own previous research (546 words), regarding the duality-of-mind approach for emotion formation. This is a first attempt at creating affective norms for Polish words. The norms are based on ratings by a total of 1,670 college students (852 females and 818 males) from different Warsaw universities and academies, studying various disciplines in equal proportions (humanities, engineering, and social and natural sciences) using a 9-point Likert Self-Assessment Manikin scale. Each participant assessed 240 words on six different scales (40 words per scale) using a paper-and-pencil group survey procedure. These affective norms for Polish words are a valid and useful tool that will allow researchers to use standard, well-known verbal materials comparable to the materials used in other languages (English, German, Portuguese, Spanish, French, Dutch, etc.). The normative values of the Polish adaptation of affective norms are included in the online supplemental materials for this article.

**Electronic supplementary material:**

The online version of this article (doi:10.3758/s13428-014-0509-4) contains supplementary material, which is available to authorized users.

The psychology of emotion is currently attracting increasing attention from numerous researchers (Kagan, 2007). The first problem in this field is to define emotions in a manner proper for empirical research that can allow one to evoke and measure them, while also finding research methods that allow for comparison of the results of different studies within one culture and between cultures (Coan & Allen, 2007). The most commonly used method is pictorial material—for example, the International Affective Picture System (IAPS; Lang, Bradley, & Cuthbert, 1999) or the Nencki Affective Picture System (Marchewka, Żurawski, Jednoróg, & Grabowska, 2014)—but not all human emotional experiences are represented in such a manner. Duality-of-mind theories (see Gawronski & Creighton, 2013), which describe and compare the automatic (heuristic) and the reflective (systematic) minds, reveal that a figurative form of material is appropriate for automatic (System 1: Kahneman, 2011) processing. However, reflective processing (System 2) is based on verbalization and language (see Rolls, 2000), and it is therefore also important to develop research methods based on verbal materials, such as affective norms for words (Bradley & Lang, 1999a; Stevenson, Mikels, & James, 2007) and further adaptations to different languages, such as European Portuguese (Soares, Comesaña, Pinheiro, Simões, & Frade, 2012), Spanish (Redondo, Fraga, Padrón, & Comesaña, 2007), French (Monnier & Syssau, 2013), German (Kanske & Kotz, 2010; Lahl, Göritz, Pietrowsky, & Rosenberg, 2009; Võ et al., 2009; Võ, Jacobs, & Conrad, 2006), Finnish (Eilola & Havelka, 2010), and Dutch (Moors et al., 2013). The Affective Norms for English Words (ANEW) list has been extended to cover an increasing number of words for different lemmas, such as the list of 13,915 English words prepared by Warriner, Kuperman, and Brysbaert (2013). All of this research was initially based on the ANEW list, since it provides a reference for the methodology and reliability of new assessments.

## Affective words’ meaning: Valence, arousal, dominance

**Fig. 1:**
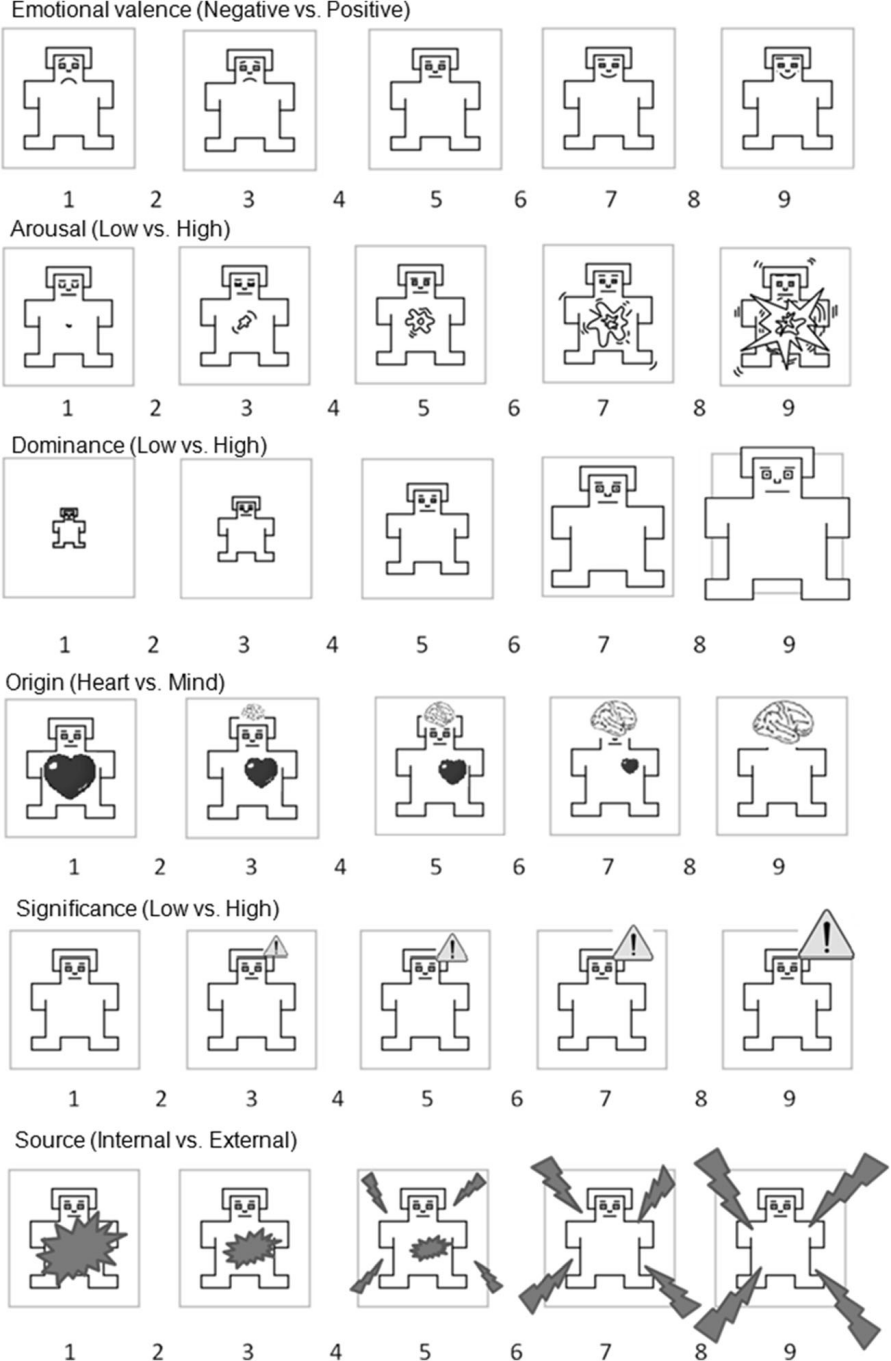
Self-Assessment Manikins (SAMs) for all scales (both classical and new proposed) in this study

Emotional words can be captured using dimensions other than the valence of emotions. Furthermore, these dimensions have a genuine impact on the processing of words. The first attempt to understand word-meaning components was made by Osgood, Suci, and Tannenbaum (1957), who used semantic differential as a tool to describe concepts, images, or sounds. Using factor analysis, they distinguished three major factors that are useful for the purposes of concept perception: valence, arousal (excitement load), and dominance (the degree of control). On this basis, Lang (1980) and Bradley and Lang (1999a) developed a pictorial scale that does not require the use of language, called the Self-Assessment Manikin (SAM). The aim of this scale was to illustrate the aforementioned three dimensions (see Fig. 1), and the ANEW (Bradley & Lang, 1999a), IAPS (Lang, Bradley, & Cuthbert, 1999), and International Affective Digitized Sounds (Bradley & Lang, 1999b) were developed using this methodology. The affective components of verbal material (valence, arousal, and dominance) shape subjective experiences (e.g., Barrett, 1998, 2004), physiological responses (e.g., Cuthbert, Schupp, Bradley, Birbaumer, & Lang, 2000; Dillon, Cooper, Woldorff, & LaBar, 2006; Fischler & Bradley, 2006; Gibbons, 2009; Schupp et al., 2000; Schupp et al., 2004), and behavioral psychological processes, such as cognitive control (e.g., Schimmack & Derryberry, 2005), remembering (e.g., Doerksen & Shimamura, 2001; Ferré, 2003; Hadley & MacKay, 2006), categorization (e.g., de Houwer, Hermans, Rothermund, & Wentura, 2002; de Houwer & Randell, 2004), and affective priming (e.g., Gibbons, 2009).

## Duality-of-mind approach in the field of emotion: Origin, source, and subjective significance

The three described dimensions do not seem to exhaust the complexity of emotional stimuli. In our studies (Jarymowicz & Imbir, in press), we postulated that two additional dimensions crucial for emotion formation should be considered: the origin (automatic vs. reflective) and source (internal vs. external) of the emotional process. With respect to origin, the main assumption is that the first type of evaluative mechanism (automatic) is based on more biologically (and evolutionarily) defined subcortical mechanisms of processing (Damasio, 1994; LeDoux, 1996, 2012; Sander, Grafman, & Zalla, 2003), whereas the second type (reflective) is connected with neocortical processes and the particular role of the orbitofrontal area (Balleine, Leung, & Ostlund, 2011; Denburg et al., 2007; Rolls, 2000; Young & Shapiro, 2011). Models of the emotional brain offer an important insight into the neurobiological nature of emotions. In particular, two brain pathways, postulated by LeDoux (1996) and Rolls (2000), leading to emotional reactions (via the limbic system or the cortex) help us to distinguish between evaluations connected with primary (prior to conscious cognition) and secondary (due to conscious cognition) affects.

This distinction is consistent with the well-known concept of “preferences,” which “need no inferences” (Zajonc, 1980). From the psychological perspective, the essence of this distinction is related to the bases of *automatic appraisals* (connected to System 1: Kahneman, 2011) versus *reflective*, *deliberative appraisals* (connected to System 2). Originally, Kahneman (2003) did not describe System 2 as emotional; in his proposition, emotions were characteristic of System 1 processing. But this brings us to a definitional question. In Kahneman’s theory, emotions are associative reactions to environmental conditions promoting heuristic and quick thinking. In fact, *automatic appraisal* is often labeled by ordinary people and many psychologists as being simply emotion. System 2 processing may result in emotional outcomes as well, often labeled as feelings, self-conscious emotions, or appraisals. *Reflective*, *deliberative appraisals* result in similar emotional responses, but are based on more complex, rule-based, and controlled processes of (a) situation construction and understanding, (b) evaluative standard (Reykowski, 1989) activation, and (c) comparison of one’s actual state to the standard resulting from emotion. System 2 processing is flexible, and the participant may decide with interpretation to choose, which standard to activate, and how crucial the comparison is.

When describing and comparing automatic emotions to reflective ones, we have to discuss the issues that contrast *automaticity* (Moors, 2013) with *automatization*. In recent years, the feature-based approach to automaticity has increasingly defined automatic processes as uncontrolled, unintentional, autonomous, goal-independent, unconscious, efficient, fast, and purely stimulus-driven. All of these features (except, in most cases, unconsciousness) are related to emotions of automatic origin (Jarymowicz & Imbir, in press). In real-life situations, it is hard to fulfill all of the criteria to say that a process is automatic. In fact, such a situation is rather rare. The other process is automatization, which may be related to both automatic and reflective appraisals. For example, some of the evaluative standards are often active and become obvious to most people (e.g., equal rights for men and women). These standards evoke automated affective responses to situations when in violation. This response is reflective in origin (i.e., based on cognitive appraisal), but immediate due to automatization. These facts could be confusing for participants on a subjective level of analysis and may be reflected in norms collection (in the case of assessment range, see the online supplemental materials).

Another important factor in the modulation of functioning could be the source of emotion (internal vs. external) with regard to a physical cause of emotions or feelings. Some experiences are reactions to internal events, processes or concepts, such as disruption of homeostasis (automatic, System 1 level) or self-standards (reflective, System 2 level). Others are reactions to external events, processes, or concepts, such as hedonic/aversive reactions (automatic, System 1 level) or beyond-self-standards, a sense of what is bad or good, without considering one’s own interests (reflective, System 2 level). Of course, everyday experience consists of many mixed states (Jarymowicz & Imbir, in press). For example, joy may be a description of a state of automatic (a tasty meal) or reflective (graduation) origins, and internal (realization of self-standards, such as fitness) or external (seeing a marvelous New Year’s Eve fireworks display) sources. One of the aims for creating norms of the two aforementioned dimensions (automatic vs. reflective) was to identify which of the 1,586 words were clearly connected to one end of each scale.

We also believe that arousal is not the only possible mechanism of activation. Excitement (arousal) is characteristic only of automatic emotions (Imbir & Jarymowicz, 2013) and processing in System 1 (Kahneman, 2011). Reflective emotions, based on conceptual mechanisms, should have their own activation mechanism that is specific to the system (see Gawronski & Creighton, 2013). We propose that it should refer to the (subjective) perception of its significance (regulative weight) for a given purpose. Activation (arousal) enhances processes that are important for survival (e.g., the fight or flight reaction) or that are System 1-specific (e.g., associative thinking or remembering in trauma; Kahneman, 2003, 2011) because of its nonspecific energy load. Arousal operates in a nonverbal bodily manner, results in physiological changes (heart rate, electrodermal activity, blood pressure, etc.), and fluctuates automatically without awareness by the participant. Analogously, System 2-specific activation should enhance processes characteristic of this type of processing. Perception of a situation as being subjectively important and significant improves motivation to invest energy resources in thinking using System 2 (rule-based) processing, which is costly. Of course, this type of activation is not as simple as arousal at a psychological level (Russell, 2003). Activation of System 2 (reflective) should work using these system-specific mechanisms and architecture of mind and in particular should engage goal relevance and future planning. To decide whether something is significant and keep it in mind are not simple tasks and are probably due to the prefrontal cortex (Damasio, 1994). Children learn to control their impulsive behavior when their prefrontal cortex matures. Education and tasks given at school focus on increasing cognitive complexity and the ability to use rule-based System 2 processing. When you decide that something is significant, you should activate your goals and keep them in mind while planning, executing, and finishing behavior. This System 2 activation should be based on a verbal and conceptual mechanism requiring energy resources. This type of activation is conscious and fluctuates in a controlled manner, on the basis of subjective decisions. To distinguish between the two types of activation, comparing stimulus processing of different origins from a duality-of-mind perspective is crucial. This may help us explain why in some cases reflective (System 2) processes are absent, and in other situations they are vivid.

Our previous findings suggest that the origins of emotions may modulate cognitive control, as assessed using the antisaccade test and the emotional Stroop test (Imbir & Jarymowicz, 2013), and other investigations have shown that sources of emotions may modulate the scope of attention (Imbir, 2013). These preliminary studies suggest that the duality-of-mind approach to emotions and its consequences for functioning are topics worthy of attention. It is impossible to answer all questions that arise when a new theoretical proposition is given without well-established research methods. In this article, the aim is to provide a list of Polish words with standard ratings of six dimensions. Three of them are classical but have never before been adapted to the Polish language, whereas three are new and were developed as an attempt to establish affective material connected with the duality-of-mind perspective (Jarymowicz & Imbir, in press) and to provide an opportunity for testing new hypotheses.

## Method

### Participants

A total of 1,670 students (852 females and 818 males) from different Warsaw universities and academies participated. They were studying various disciplines in equal proportions (humanities, engineering, and social and natural sciences), and ranged in age from 18 to 49 years (mean [*M*] = 20.74 years, standard deviation [*SD*] = 2.77) Approximately 120 additional participants did not contribute due to leaving the study (they did not provide more than two answers, omitted one scale, gave one answer for more than ten words in a row, or finished in under 10 min) or to not being native Polish speakers. The ratings were obtained between October 2013 and February 2014.

### Materials and procedure

We assessed a list of 1,586 Polish words obtained from two sources. The first was a translation to Polish of 1,031 words from the original ANEW list (Bradley & Lang, 1999a). The translation was based on the Google translation engine, the Cambridge Dictionary of English, and the PWN Oxford English–Polish Dictionary. First, all ANEW words were translated and back-translated by the Google translation engine. We found congruent translation for 799 words. Then two bilingual persons checked the machine translations to indicate whether the translation was correct or provide their own suggestions. At this stage, we found an additional 54 incongruent words in their assessments. In the case of ambiguous translations and incongruent words (*N* = 276), we also used the help of a professional philologist specialized in the English language and with a deep knowledge of American culture, resulting in 1,040 Polish words. Some of the words have more than one meaning in Polish: 14 have two meanings, six have three meanings, and one has four meanings. Also, some Polish words have more than one English translation: 65 have two translations, and two have three translations (see also the supplemental materials). We added 546 words from our own list used in previous research, which was compiled to elicit emotions of different origins and sources (see Jarymowicz & Imbir, in press). The translation procedure was similar for the additional Polish words (machine translation and back-translation, correction, and professional help in the case of 279 ambiguous words), resulting in a list of 1,586 words. The number of letters per word varied between two and 19 (*M* = 7.94, *SD* = 2.77). Frequency of usage was taken from a frequency list of Polish words prepared using an electronic text database (Kazojć, 2011), and the frequency varied from 0 to 100,352. This estimation was based on a vast collection of electronic Polish language texts (books, web pages, newspapers, magazines, etc.), and the number of repetitions of single words in the entire collection was considered (see Kazojć, 2011). Considering all 1,586 words, some (82) had an ambiguous meaning that could be translated into different English words: 80 into two words, and two into three words. Some Polish words (122) could be translated into either one or more English words: 108 into two words, 13 into three words, one into four words. The prepared list consisted of 1,198 nouns, 97 verbs, 260 adjectives, 3 adverbs, and 28 other items (mainly two-word phrases).

A list of 1,600 words (14 words were doubled in order to check the reliability of assessments; see below) was randomly arranged in two different orders, to minimize the impact of the surrounding words on the ratings. Both orders were divided into 40 sublists, each containing 40 words. We prepared six SAM scales, three of which (valence, arousal, and dominance) were adapted from Lang (1980), whereas we created the remaining three (origin, significance and source) specifically for this research. The latter three dimensions correspond to a taxonomy of human emotions (Jarymowicz & Imbir, in press) distinguishing between automatic and reflective origins of human affective states. Figure 1 illustrates the SAM scales we used in this study.

Each participant rated six different lists containing 40 words, one for each scale (this totaled 240 words for each participant). We prepared 80 counterbalanced versions of a paper-and-pencil questionnaire in order to avoid surrounding-word effects. These versions were also used to assess reliability. Each SAM scale was preceded by a description of the dimension with examples of the scale-end states (see Table 1). This was done both to clarify the meaning of the SAM pictures and to address an intuitive tendency to describe emotional states and feelings on the valence dimension (as positive or negative) only. Therefore, the examples of five descriptions presented negative, as well as positive, feelings—for example, “The last picture (excitement) shows an individual who is bursting with arousal—relevant states could include excitation, euphoria, excitement, rage, agitation, or anger.”

**Table 1:** SAM scales descriptions

Valence of experiences: Negative vs. positive *Znak doznań: Negatywny kontra Pozytywny*	The first picture shows a person who is clearly distressed—relevant experiences could include panic, irritation, disgust, despair, defeat, or crisis. The last pictures shows an individual who is obviously elated—relevant experiences could include fun, delight, happiness, relaxation, satisfaction, or repose. The remaining pictures depict intermediate states.
Intensity of experiences: Tranquillity vs. excitation *Intensywność doznań: Spokój kontra Ekscytacja*	The first pictures shows an individual who is very calm, almost sleeping—relevant states could include relaxation, tranquility, idleness, meditation, boredom, or laziness. The last picture shows an individual who is bursting with arousal—relevant states could include excitation, euphoria, excitement, rage, agitation, or anger.
Sense of dominance: Being under control vs. controlling *Odczucie dominacji: Bycie pod kontrolą kontra Kontrolowanie*	The first picture shows an individual who feels a lack of control and agency—relevant states could include subordination, intimidation, subjugation, withdrawal, submission, or resignation. The last picture shows a person who is dominant and in control of the situation—relevant states could include control, influence, being important, dominant, recognized, or decisive.
Origin of experience: From heart vs. reason *Pochodzenie doznań: z Serca kontra Rozumu*	The first picture shows an individual who is overwhelmed with appeals from the heart—words that could represent these experiences include being beside oneself, complete commitment, full engagement, impulsivity, spontaneity, lack of hesitation. The last picture shows a person who is under the sway of the mind, who is reflective—words that could be used to represent this state include feelings that result from contemplation, planning, consideration, prediction, choices, or comparisons.
Significance of experience: Insignificant vs. Significant for the individual *Waga doznań: Nieważne kontra Ważne dla człowieka*	The first picture shows a person whose current experience is not significant to his goals, plans, and expectations—his experience could be referred to using words such as trivial, gone unnoticed, fleeting, inconsequential, insignificant, unimportant. The last picture shows a person who is experiencing something very important to his goals, plans, and expectations—his experience could be referred to with words such as vitally important, significant, turning-point, consequential, meaningful, decisive.
Source of experiences: Internal vs. External *Źródło doznań: Wewnętrzne kontra ze Środowiska*	The first picture shows a person who is afflicted by experiences springing from, having their roots, in his insides—these experiences could be represented with words such as hunger, thirst, pain, self-loathing, self-acceptance, pride. The last picture shows a person who perceives and experiences stimulation from the outside—these experiences could be represented with words such as delight in nature, vacation, carrion, democracy, human well-being, injustice.

The participants assessed each word on a 9-point Likert scale on which 1 meant *negative/calm/being in control/from the heart/of no consequence/internal*, and 9 meant *positive/excited/controlling/from the mind/important/external*. In all SAM scales, 5 was described as a *neutral/mixed/moderate state*.

All participants assessed words during the paper-and-pencil procedure, administered as collective sessions in seminar rooms (with 15 to 80 participants in each session) after regular courses in different departments. In each session, before data collection we described the aim of the study to the participants. We emphasized the voluntary and unpaid nature of their participation, as well as the confidentiality of the results. We explained each SAM scale and gave examples. We answered all questions concerning the instructions, SAM descriptions, and the task itself. Participants were not instructed what to do with regard to ambiguous words, so this might be reflected in the variability of ratings. In the event of an absence of familiarity with the words, the participants were instructed to leave an empty space. After the assessment, the participants were asked to complete a socio-demographic questionnaire (e.g., gender, age, and department). The entire procedure took approximately 25 min. There was no time limit, but participants were encouraged to answer as quickly as possible following their first impression and intuition. Each word in the data set was rated by 38 to 44 participants (*M* = 41.69, *SD* = 0.96). The 14 doubled words were rated by 80 to 85 participants (*M* = 83.46, *SD* = 1.17).

## Results

We conducted the following analyses. First, the data were entered into the database. Data collected from participants who left the study (see above) were excluded and did not appear in the database (approximately 120 questionnaires). We then calculated the mean, *SD*, and range (min and max value) for each word. The supplemental materials include all values of valence, arousal, dominance, origin, significance, and source assessments. All words have their Polish form, English translation (some have more than one translation), part of speech (N = noun, V = verb, A = adjective, X = adverb, I = other—e.g., phrases consisting of two words), number of letters, and frequency estimation in the Polish language. All analyses were carried out using the IBM SPSS 21 statistical package.

**Fig. 2:**
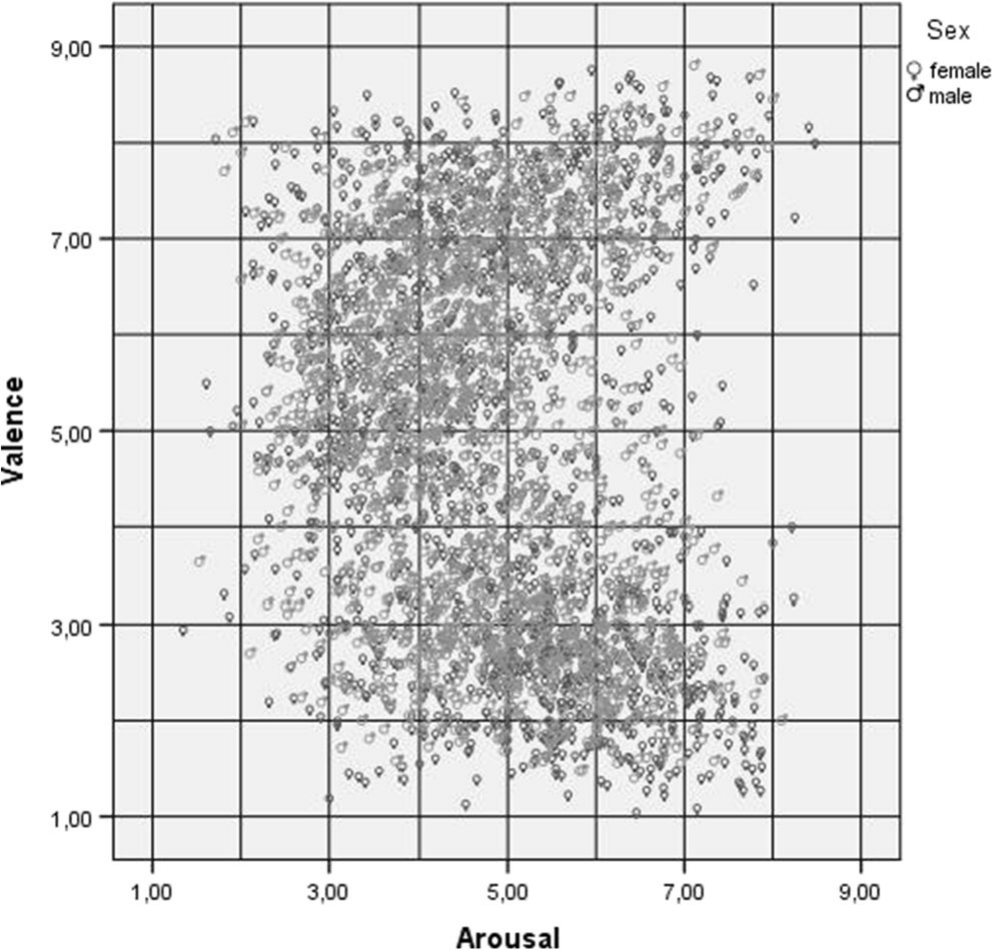
Distribution of mean values (for males and females) for 1,586 Polish words in the valence and arousal affective dimensions

Figure 2 shows the typical boomerang shape of the distribution of the ratings of 1,586 Polish words in the bidimensional (Valence × Arousal) affective space, for both males and females. This shape has been observed in the norms of Bradley and Lang (1999a), as well as in several adaptations, to Spanish (Redondo et al., 2007), Portuguese (Soares et al., 2012), and (partially) a new list creation (Moors et al., 2013).

Table 2 presents descriptive statistics (*M*s, *SD*s, and ranges) for all affective variables (scales) used, as well as for the linguistic variables. Statistics were calculated for the total sample and for both genders separately.

**Table 2:** Summary of variables included in the word list, with means (*M*s), standard deviations (*SD*s), and ranges for all participants and for females and males separately

Affective Dimension	All			Females			Males		
	*M*	*SD*	Range	*M*	*SD*	Range	*M*	*SD*	Range
Valence	4.87	1.96	1.29–8.67	4.84	2.11	1.05–8.76	4.9	1.87	1.48–8.80
Arousal	4.83	1.22	1.66–8.07	4.92	1.35	1.58–8.48	4.74	1.21	1.52–8.10
Dominance	4.85	1.21	1.71–8.45	4.85	1.35	1.59–8.73	4.85	1.22	1.80–8.42
Origin	4.79	1.06	1.78–8.21	4.78	1.18	1.45–8.35	4.8	1.07	1.88–8.16
Significance	4.97	1.21	1.60–8.35	4.98	1.36	1.50–8.70	4.97	1.17	1.68–8.48
Source	4.75	0.89	2.38–7.42	4.74	1	2.11–7.47	4.75	0.95	2.11–7.8
Frequency	1,838	5,972	0–100,352						
Number of letters	7.95	2.77	2–19						

### Reliability

In order to check the reliability of the scales used to measure the six dimensions, we applied three different methods. The first of these was estimation by the split-half method, in which the list of all words (1,600) was randomly and equally divided into two different orders, each of which was further divided into 40 sublists, distributed over scales in 40 questionnaire versions (each word in each scale appeared with different surrounding words in versions A and B). The mean and *SD* were calculated for each word, and these were compared using Pearson’s correlations. Table 3 (first column) presents the correlations obtained between both halves of the assessments. All were significant at *p* < .001. The correlations varied from .95, for valence, to .62, for source.

**Table 3:** Reliability estimations for each variable: (a) Split-half correlations (Pearson’s *r*) estimated for all words, (b) correlations (Pearson’s *r*) with a 96-word pilot assessment, (c) number of significant differences between 14 doubled words (*t* test), and (d) correlations for doubled words (Pearson’s *r*)

Scale	Split-Half Correlations	Correlations With 96-Word Pilot-Study Assessments	*t* Tests for 14 Doubled Words (*N* of sign.)	14 Doubled Words Correlations
Valence	.95	.99	0	.99
Arousal	.78	.90	0	.97
Dominance	.78	.88	0	.90
Origin	.73	.81	0	.90
Significance	.78	.90	1 (*zimno*)	.91
Source	.62	.70	1 (*skorpion*)	.77

The second method to establish scale reliability was estimation by comparison with a previous study based on ratings of a list of 120 Polish words (Imbir, 2014). These ratings had been performed by 79 female undergraduate psychology students from 20 to 25 years of age (*M* = 22.1, *SD* = 1.3). Each word was rated by 25 to 28 participants and served as a pilot stage for the present study. The assessments of the psychology students were not included in the presented data set (Imbir, 2014). Of those 120 words, 96 were included in the present list. To estimate reliability, we calculated Pearson’s correlations between the ratings obtained in the pilot stage and those of the present study, which are shown in Table 3 (second column). All were significant at the level of *p* < .001, varying from .99, for valence, to .70, for source.

The third method was estimation carried out after adding the 14 words duplicated from the list of 1,586 words. These were “acceptance” (*akceptacja*), “ecstasy” (*ekstaza*), “sin” (*grzech*), “rape” (*gwałt*), “anxiety” (*lęk*), “pity/mercy” (*litość*), “passion” (*pasja*), “flood” (*powódź*), “fire” (*pożar*), “respect” (*respekt*), “scorpion” (*skorpion*), “cuddle/snuggle” (*tulić*), “health” (*zdrowie*), and “cold” (*zimno*). We choose random words for duplication to avoid similarities in their meanings. The words were spread across the two versions of the questionnaire and assessed by different participants. To estimate reliability, we used *t* tests and Pearson’s correlations between the first and second appearances of the word in each scale used. Table 3 presents the numbers of significant (*p* < .05) *t* tests (third column) and correlations between the first and second appearances (fourth column). Almost all *t* tests were not significant, with the exception of a single difference in the Significance scale for the word “cold” (*zimno*), *t*(81) = 2.23, *p* = .028, and a single difference in the Source scale for the word “scorpion” (*skorpion*), *t*(82) = 2.85, *p* = .006. Correlations varied from .99, for valence, to .77, for source, and all were significant at the *p* < .001 level.

### Correlations between variables

Pearson’s correlations were calculated for all affective variables (valence, arousal, dominance, origin, significance, and source) and linguistic variables (natural logarithm of frequency, number of letters) for all words and for the ANEW words only. Almost all correlations were similar in the 1,586-word set and in the list of 1,040 ANEW words, and are shown in Table 4.

**Table 4:** Correlations between the variables (Pearson’s *r*)

		Arousal	Dominance	Origin	Significance	Source	Frequency (LN)	Number of Letters
Valence	All words	–.15^**^	.64^**^	–.07^**^	.46^**^	.09^**^	.18^**^	–.04
	ANEW	–.06	.64^**^	–.11^**^	.4^**^	.16^**^	.15^**^	–.03
Arousal	All words		.21^**^	–.2^**^	.24^**^	–.07^**^	–.02	.08^**^
	ANEW		.29^**^	–.22^**^	.36^**^	–.12^**^	–.007	.11^**^
Dominance	All words			.15^**^	.5^**^	.1^**^	.13^**^	.007
	ANEW			.06^*^	.46^**^	.13^**^	.17^**^	–.01
Origin	All words				–.08^**^	.2^**^	–.03	–.03
	ANEW				–.16^**^	.25^**^	–.003	–.06^*^
Significance	All words					–.25^**^	.24^**^	.12^**^
	ANEW					–.25^**^	.26^**^	.1^**^
Source	All words						.05	–.22^**^
	ANEW						.02	–.15^**^
Frequency (LN)	All words							–.46^**^
	ANEW							–.38^**^

^**^ *p* < .001, ^*^ *p* < .05

Here, we describe only correlations of *r* > .35 or that are important from a theoretical perspective. Valence weakly negatively correlated with arousal with regard to the larger word set (*r* = –.15), and did not correlate for the ANEW words. Considering the classical boomerang shape of the ratings distribution in the bidimensional (Valence × Arousal) affective space (see Fig. 2), we may say that the relationship between valence and arousal in Polish is the same as it is around the world. To check whether the correlations were different for negative and positive words, with the suggested boomerang shape distribution, we conducted separate analyses of correlations for both groups of words. Participants’ assessments were the criterion of division. Words assessed as equal to or less than 5 on the valence dimension were treated as negative (*N* = 810), whereas those given a rating of more than 5 were regarded as positive (*N* = 776). The linear Pearson’s correlation for negative words appeared to be significant (*p* < .001) and was negative (*r* = –.40). The same correlation for positive words also appeared to be significant (*p <* .001) and was positive (*r* = .36).

Valence had a strong positive correlation with dominance (*r* = .64) for both word groups. This means that the participants rated positive experiences as controllable, and rated negative experiences as uncontrollable. Origin had weak correlations with all of the classical dimensions (valence, arousal, and dominance), which may suggest the independence of this scale. Subjective significance was positively correlated with valence (*r* = .46 or .4) and dominance (*r* = .5 or .46). This means that the participants rated positive and controlled experiences as being more subjectively significant and important than negative and uncontrolled experiences. This corresponds with the expectation that mental substitute of activation should be more sensitive to goal-congruent situations and experiences (positive and controlled). Number of letters had a negative correlation with the natural logarithm of word frequency. This means that long words are less frequent than short ones in Polish.

### Correlations between variables in different adaptations of affective norms

It is worth comparing the correlations obtained with the ANEW data set (Bradley & Lang, 1999a). They were similar for arousal–valence and dominance–valence, but slight differences were observed with regard to the arousal–dominance correlations (Table 5). The correlations obtained in the present study also have similarities with the findings of Warriner et al. (2013), particularly with regard to the high positive correlation between dominance and valence. Surprisingly, the arousal–dominance correlation was negative in this 13,915 word list, whereas it was positive in all another studies. Comparing our results to those of Moors et al. (2013), who studied norm creation for Dutch words, we found bigger differences, particularly with regard to the low positive dominance–valence correlation and the relatively high positive arousal–dominance correlations. This may suggest cultural differences in the understanding of words by the Dutch participants in the Moors et al. study. Table 5 presents correlations with the different ANEW adaptations.

**Table 5:** Comparison between dimension correlations (Pearson’s *r*) in different studies of affective norms

	Present Study: All / ANEW	Bradley & Lang, 1999a	Warriner et al., 2013	Moors et al., 2013
Arousal and valence	–.15 / –.06	–.046	–.185	–.01
Dominance and valence	.64 / .64	.839	.717	.27
Arousal and dominance	.21 / .29	.072	–.180	.59

## Discussion

We collected word norms for 1,586 Polish words for the classical affective variables—valence, arousal, and dominance—and for new variables connected with the taxonomy of human emotion (Jarymowicz & Imbir, in press): origin, significance, and source. The latter three dimensions correspond to the duality-of-mind perspective (see Gawronski & Creighton, 2013), describing automatic and deliberative mental processes. Comparing the results of the international adaptations of ANEW (and wider word list construction), we may conclude that there is only one universal relationship between the three classical dimensions: a boomerang-shaped Valence × Arousal distribution. This shape was also obtained with our sample, which may suggest the validity of classical-dimension ratings.

### Reliability and proposed dimension status

We observed a consistent pattern of reliability estimations, regardless of the method used. This suggests that reliability estimation is itself reliable. The second issue was the status of the dimensions measured: classical and newly proposed. All estimations showed almost perfect reliability for valence (*r* = .95 or .99). This is not surprising, considering the intuitive status of valence when speaking of emotions and feelings on an everyday level.

The other three dimensions had slightly lower, but still very satisfactory, reliability estimations. With regard to arousal assessments, estimations varied from *r* = .78 to .97, whereas estimations for dominance varied from *r* = .78 to .90, and estimations for subjective significance varied from *r* = .78 to .91. This suggests that the method used to measure those scales is reliable, and that the results can be widely used by researchers interested in emotion, word perception, and word processing.

Reliability estimations a bit lower were found with regard to the origin scale (*r* from .73 to .90), especially with regard to the split-half estimation. An increasing number of scientists (e.g., Kagan, 2007; Russell, 2003) insist on searching for the scientific language and basic mechanisms underlying emotional processing. However, the underlying processes may be difficult to find on a subjective level of analysis. Another explanation is the automatization of some affective processes that are reflective in their origin, providing some additional confusion for participants. To make it easier to access the state of origin, we used the dichotomy of heart versus mind, which is well-known in Western culture and has become extremely popular in Poland in the last 3 years through a series of funny TV commercials. This popularity itself may have influenced assessments (e.g., by activating stereotypes); however, it could be a useful hint for participants.

The worst reliability estimations were found for the source dimension (*r* from .62 to .77). This may suggest that this dimension is not at all intuitive and that the participants had difficulties assessing this quality of their experience. Emotions are treated as internal (bodily) experiences, so it was hard for them to answer and say that something was external. It is worth paying attention to the relatively small range found with this scale (2.38–7.42), which suggests difficulties in making the assessments. Therefore, this scale is somewhat biased, and our analyses do not confirm its reliability for use. On the other hand, we have some unpublished data concerning short texts suggesting that the scale itself is reliable, but the pure context provided by the word may reflect the rather low reliability estimations for this scale.

### Validity of proposed dimensions

Validity estimation for a new proposed dimension is not simple. Our first attempt was made in order to check the correlation pattern of the assessments (see Table 4). The correlation analyses showed very low correlations of the origin and source dimensions with valence, arousal, and dominance. This indicates that origin and source are distinct constructs. We also found a low correlation of significance with arousal and origin. This suggests that the three new proposed activation-type dimensions may, in fact, be based on different mechanisms—namely goal congruence and rule-based processing.

A second attempt might be made in cases in which this material is used in experimental work. This still needs to be done in the present case, but some preliminary data suggest that the origin and source of presented affective materials shape cognitive processes, making it difficult to maintain cognitive control in cases of automatic related-material presentation (Imbir & Jarymowicz, 2013), as well as to broaden the scope of attention in cases of reflective and external material presentation (Imbir, 2013). We think that all three of these new dimensions bring a fresh light to the emotional content of word understanding.

### Possible use of ANPW and description of the database

These affective norms of 1,586 Polish words are important for the development of affective research, especially in Polish-speaking samples. This has been the first attempt to address this issue in the literature. However, recently, another set of affective norms was introduced for Polish words (NAWL: Riegel et al., 2014), including valence, arousal, and imageability ratings for 2,902 words taken from the Berlin Affective Word List (Võ et al., 2009).

ANPW, due to its three new proposed dimensions, allows researchers to test new hypotheses in the field of affective sciences’ duality-of-mind approach. This database provides six affective dimension ratings used across all words. Hopefully the present study will be useful for researchers interested in emotions and the meanings of words. It may help them to plan research and choose effective experimental materials, particularly in neuroimaging (EEG, fMRI) studies. This is especially true when complex emotions are of interest, or when complex emotions are compared to simple emotional states. Another field in which the results of this study may be used is semantic priming; these materials may be presented in a degraded manner, and the consequences may be measured. The third area includes lexical and language studies. The materials may be used in passive-reading or classification procedures, or could be the basis for active procedures, such as remembering in response to single-word situations or from one’s own life.

The normative values of the Polish adaptation of affective norms are included in the online supplement to this article. In the first column, the full list of Polish words (1,586) and their English translations are provided. Then, three lexical variables (part of speech, number of letters, and frequency of appearance in the Polish language), as well as the six affective dimensions (valence, arousal, dominance, origin, significance, and source) are reported. For each affective variable, the mean and *SD* are presented in the “short” spreadsheet. Additional data referring to the number of participants assessing a single word [*N*] and the range, represented by the minimal [*Min*] and maximal [*Max*] rates, are presented in the “full” spreadsheet. In each case, the ratings are presented for all participants together, as well as for females and males separately. The last columns in each spreadsheet present the number of possible English translations for a single Polish word, as well as the reference numbers for the English translations in the ANEW data set. The ANPW is freely available to the scientific community for noncommercial use, in the form of supplemental online material hosted by the Psychonomic Society.

## Electronic supplementary material

Below is the link to the electronic supplementary material.

ESM 1 (XLS 1.85 MB)
